# Dietary Energy and Protein Levels During the Prelay Period on Production Performance, Egg Quality, Expression of Genes in Hypothalamus-Pituitary-Ovary Axis, and Bone Parameters in Aged Laying Hens

**DOI:** 10.3389/fphys.2022.887381

**Published:** 2022-04-28

**Authors:** Qian Xin, Ning Ma, Hongchao Jiao, Xiaojuan Wang, Haifang Li, Yunlei Zhou, Jingpeng Zhao, Hai Lin

**Affiliations:** ^1^ Shandong Provincial Key Laboratory of Animal Biotechnology and Disease Control and Prevention, College of Animal Science and Veterinary Medicine, Shandong Agricultural University, Tai’an, China; ^2^ College of Life Sciences, Shandong Agricultural University, Tai’an, China; ^3^ College of Chemistry and Material Science, Shandong Agricultural University, Tai’an, China

**Keywords:** bone quality, egg quality, energy, gene expression, protein

## Abstract

Nutrition during the pre-lay period takes effect on the production performance in the laying flock. This study evaluated the effects of dietary energy and protein levels in pre-lay diet on performance during the whole laying period and the egg quality, bone quality, and mRNA expression of hypothalamus-pituitary-gonadal (HPG) axis-related genes of hens at the end of the laying cycle. A total of 1,856 15-wk old Hy-Line brown pullets were randomly assigned to one of the four dietary treatments: using a 2 × 2 factorial arrangement with 2 energy levels (2,700 and 2,800 kcal/kg ME, respectively) and 2 protein levels (15 and 16.5% CP, respectively). Pullets were fed *ad libitum* from 15 to 20 wk and from 20 wk onward, fed with a similar laying diet till 72 wk of age. At 72 wk, the expression of genes in the hypothalamus, pituitary, ovarian, and follicles and bone quality was evaluated. At 72wk, there were no differences in production performance, BW, organ index, and ovarian parameters among the dietary treatments. High-CP diet increased the egg shape index and eggshell thickness (*p* < 0.05), but the eggshell breaking strength, Haugh unit, and albumen height did not differ among the treatments. Neither dietary energy nor protein level took an effect of bone quality. Low-energy diet increased the mRNA expression of gonadotropin-releasing hormone-1 (*GnRH-1*) in the hypothalamus (*p* < 0.05). The mRNA expression level of estrogen receptor-1 (*ESR-1*) in the hypothalamus and ovary was elevated by the 2,700 ME-15%CP diet (*p* < 0.05). The expression of cytochrome family 17 subfamily A polypeptide 1 (*CYP17A1*) in the large white follicle (LWF), small yellow follicles (SYF) and dominant follicle (DF) was decreased by the 2,800 kcal/kg diet (*p* < 0.05). These results indicate that the prelay diet had no influence on the production performance but had minimal effect on the eggshell characteristics and bone parameters. These results suggest that the energy and protein level of the prelay diet changes the expression of HPG axis-related genes of hens around the end of the laying cycle without changing the circulating sex hormone profile. The effect of prelay diet on the endocrinal adjustment at the end of the laying cycle needs to be investigated further.

## Introduction

Dietary manipulation during the prelay period can be an effective way of maintaining flock uniformity and enhancing production performance in the laying flock. The development of secondary reproductive organs and growth of ovarian follicles is thought to be between 4 and 6 wk before the first egg (16–18 wk of age) (Cave, 1984), and rapid physiological changes occur during the prelay phase (Sujatha et al., 2014). The diet’s energy and protein content must be adjusted to ensure that hens consume enough nutrients needed to cope with growth and onset of egg production (Bain et al., 2016). Preliminary evidence has demonstrated that the increase of energy and protein levels in the prelay diet increases production performance in laying hens (Lilburn and Myers-Miller, 1990; Sujatha et al., 2014). Cave (1984) reported that a high-protein prelay diet could remarkably increase egg production in laying hens. However, little attention has been given to this critical stage in the management of laying hens.

It is known that an increase in egg size is associated with an increase in dietary protein intake during the production phase (Gunawardana et al., 2008). However, the protein intake needed for development during the prelay phase and maintaining persistency in lay is unknown. Furthermore, earlier studies have reported that dietary energy intake restriction delays the sexual maturity of broiler breeders and layers (Isaacks et al., 1960; Hollands and Gowe, 1961; Fuller and Dunahoo, 1962; Gowe et al., 1965; Fuller et al., 1969). However, due to constant selection and genetic improvement, the nutrient requirement should be continuously optimized (Han et al., 2017; Saxena et al., 2020).

In birds, like other vertebrates, the hypothalamic-pituitary-gonadal (HPG) axis governs egg production by regulating the cyclic production of gonadotropins [follicle-stimulating hormone (FSH) and luteinizing hormone (LH)] and steroid hormones and the selection of a dominant follicle for ovulation (Mikhael et al., 2019). In the hypothalamus, gonadotropin-releasing hormone (GnRH) and gonadotropin inhibitory hormone (GnIH) act on the pituitary gland to either stimulate or inhibit the production of gonadotropins, respectively (Tsutsui et al., 2000; Bédécarrats et al., 2009; Bain et al., 2016). GnRH stimulates the pituitary gland to synthesize FSH and LH, which stimulates the developed gonads to synthesize sex steroids hormones for initiating sexual maturity (Tsutsui et al., 2000; Bain et al., 2016). But, GnIH inhibits the synthesis of FSH and LH. A recent research has shown that high energy diet activated the HPG axis and stimulated the secretion of reproductive hormones in broiler breeder pullets (Hadinia et al., 2020). However, little is known on the effect of prelay dietary energy and protein level on hormone levels.

Egg production performance of modern laying hens has improved significantly during the last decade. Prolonging the laying period of laying hens has become a new breeding goal, that is, the “long life” layer, which will be capable of producing 500 eggs in a laying cycle of 100 weeks (Bain et al., 2016; Gautron et al., 2021). Osteoporosis is a major and prevalent welfare condition in laying hens caused by bone loss due to absorption of structural and medullary bone for calcium needed for eggshell production (Webster, 2004). Osteoporosis has led to increased fracture, especially during depopulation and transportation of spent-layers, causing carcass condemnation (Rath et al., 2000; Webster, 2004). In laying hens, protein-energy enrichment diets may help maintain bone mass and bone strength prior to sexual maturity (Rath et al., 2000). Previous studies had demonstrated that bone quality achieved before sexual maturity lasted through the production phase and was maintained in aged laying hens (Casey-Trott et al., 2017).

We hypothesized that manipulating energy and protein levels during the prelay period will improve production performance, maintain the persistency in lay with quality eggs, and maintain bone quality until the end of the first laying cycle. Therefore, this study aimed to evaluate the effects of dietary energy and protein levels fed during the prelay period on performance, egg quality, expression genes in the HPG axis, and bone quality of aged laying hens.

## Materials and Methods

Approval for this study was granted by the Institutional Animal Care and Use Committee of Shandong Agricultural University prior to the commencement of the trial.

### Experimental Design and Animal Management

A total of one thousand eight hundred and fifty-six 15 wk Hy-Line Brown pullets were used for this trial. The experiment was a 2 × 2 factorial arrangement with two dietary energy levels (2,700 vs. 2,800 kcal/kg ME) and two dietary protein levels (15 and 16.5% CP). Each treatment was replicated 4 times with 116 pullets per replicate, all the rearing facilities were kept the same. The birds were reared in cages with 2 birds per cage. The dimensions of each cage were 45 × 35 × 35 cm (length × width × height) and equipped with a nipple drinker and feeding trough. The room temperature was maintained at 20 ± 1°C throughout the trial. Light was maintained at 10 h/d at 15 wk of age and increased gradually with 1 h/wk till 16 h/d at 30 wk. Pullets were fed experimental diets from 15 to 20 weeks of age and fed the same laying diet till the end of the experiment. All birds had free access to water and feed throughout the trial period. The formulated diets and nutrient compositions are presented in Table 1. The same batch of corn, soybean meal, bran and oil was used throughout the experiment to ensure the consistency of feed composition throughout the experiment.

**TABLE 1:** Experimental diets and nutrient compositions.

Energy	Treatments				
	2,700 kcal/kg		2,800 kcal/kg		Laying
Protein	15%	16.5%	15%	16.5%	
Corn	63	61	60	60	64
Soybean meal	17	22	17	22.5	23
Oil	-	-	2	1.5	1
Bran	15	12	16	11	-
Limestone	1.75	1.75	1.75	1.75	9.4
Choline chloride	0.07	0.07	0.07	0.07	0.07
Mono-dicalcium phosphte	0.6	0.6	0.6	0.6	0.8
Salt	0.3	0.3	0.3	0.3	0.32
Medical Stone	1.96	1.96	1.96	1.96	0.94
Lysine	0.1	0.1	0.1	0.1	0.16
Methionine	0.08	0.08	0.08	0.08	0.16
Vitamin premix	0.04^a^	0.04^a^	0.04^a^	0.04^a^	0.05^b^
Mineral premix	0.1^c^	0.1^c^	0.1^c^	0.1^c^	0.1^d^
Nutrient composition					
Crude protein^e^	15.07	16.86	14.89	16.82	15.60
Metabolizable energy, Kcal/kg	2,700	2,700	2,800	2,800	2,700
Ca, %	0.89	0.89	0.89	0.89	3.57
Available P, %	0.55	0.55	0.55	0.55	0.48
Lysine, %	0.80	0.80	0.80	0.80	0.85
Methionine, %	0.32	0.32	0.32	0.32	0.41

a Provided per kg of premix: vitamin A, 180,000 IU; vitamin D, 60,000 IU; vitamin E, 500 mg; vitamin K, 50 mg; vitamin B1, 50 mg; vitamin B2, 120 mg; vitamin B6, 60 mg; pantothenate, 200 mg; nicotinamide, 600 mg; folic acid, 16 mg; biotin, 4 mg.

b Provided per kg of premix: Cu, 0.24 g; Fe, 1 g; Mn, 2.00 g; Zn, 1.80 g; I, 13 mg; Se, 5.60 mg; Co., 10 mg.

c Provided per kg of premix: vitamin A, 230,000 IU; vitamin D, 75,000 IU; vitamin E, 500 mg; vitamin K, 86 mg; vitamin B1, 60 mg; vitamin B2, 150 mg; vitamin B6, 75 mg; vitamin B1, 1 mg; pantothenate, 200 mg; nicotinamide, 750 mg; folic acid, 35 mg; biotin, 4 mg.

d Provided per kg of premix: Cu, 300 g; Fe, 1.5 g; Mn, 2.4 g; Zn, 2.3 g; I, 20 mg; Se, 10 mg; Co., 12 mg.

e Measured value.

### Production Performance

All eggs laid were counted and weighed daily starting from the first egg until the end of the experiment (72 wk). Egg production and egg weight were recorded daily; egg mass was calculated by multiplying the egg production by the egg weight.

### Egg Quality

At 72 wk, 20 eggs per replicate were randomly selected for egg quality analysis. Egg length (mm) and width (mm) were measured using a digital vernier caliper, and egg shape index was calculated by dividing the egg length by the egg width. Eggshell thickness (mm) was measured at the equator, sharp, and blunt end of the egg using an eggshell thickness gauge (ROBOTMATION, Japan). Egg Shell breaking strength was determined using eggshell force gauge (ROBOTMATION, Japan). Haugh unit, yolk color, and albumen height were measured using Egg Multi Tester (EMT-5200, ROBOTMATION, Japan).

### Sample Collection

At the end of the experiment (72 wk), thirty-two hens (2 per replicate) were randomly selected for analysis. Blood samples were collected by venipuncture via the brachial vein into a non-coated vacutainer tube. Subsequently, blood was centrifuged at 3,500 *g* for 10 min at 4°C, and serum was recovered and stored at -20°C till further analysis.

After the blood sample was obtained, the hens were sacrificed and dissected, and the hypothalamus, pituitary, and follicles with average sizes [dominant follicles; small yellow follicles (SYF); and large white follicles (LWF)], the yolk was drained from each follicle and the follicle was everted to collect the theca interna layer and the remaining theca externa layer was removed. Tissue samples were immediately snap-frozen in liquid nitrogen and stored at −80 °C for RNA extraction. The breast muscle (BM; total of pectoralis major and pectoralis minor), leg muscle (LM; total leg muscle of the left leg), liver, abdominal fat pad, ovary, oviduct, magnum, and eggshell gland were excised and weighed. The left tibia and femur were dissected, cleaned of all adhering muscles and connective tissue, and then weighed to obtain the absolute weight. The total length and diameter of the tibia and femur were measured using a digital vernier caliper. Tibia and femur length was defined as the distance between the proximal and distal epiphysis. The tibia and femur diameter was measured at the mid-diaphysis at approximately 50% of the bone length. The tibia and femur were then kept in individual labeled zip-lock plastic bags and stored at −20°C till further analysis. The relative bone, muscle, and organ weight was calculated and expressed in percentage relative to the live body weight.

### Measurement of Serum Hormones and Biochemical Parameters

The concentrations of calcium (Ca), phosphorus (P), and activity of alkaline phosphatase (ALP) in serum were measured using Hitachi L-7020 fully automatic biochemical analyzer (Tokyo, Japan) and commercial kits (Jiancheng Bioengineering Institute, Nanjing, China). Serum concentrations of estradiol (E2), FSH, LH, and progesterone (P4)were determined by RIA using kits obtained from Union Medical & Pharmaceutical Tech (Tianjin, China). The E_2_ sensitivity of the assay was 1.4 Pg/ml, the FSH sensitivity of the assay was 0.46 mIU/ml, the LH sensitivity of the assay was 0.4 mIU/ml, the P_4_ sensitivity of the assay was 0.05 ng/ml, and all samples were included in the same assay to avoid intra assay variability. The intra assay coefficient of variation was less than 10%.

### RNA Extraction, cDNA Synthesis, and Quantitative Real-Time PCR

Total ribonucleic acid (RNA) was extracted from the hypothalamus, pituitary, ovary, and follicles using the NcmZol reagent (NCM Biotech), following the manufacturer’s instructions. The quantity and quality of RNA were measured using a NanoDrop Spectrophotometer (DeNovix). Subsequently, complementary deoxyribose nucleic acid (cDNA) was synthesized using PrimeScript RT Master Mix (TaKaRa Biotechnology). The primers were designed using Primer 5.0 software (SPS Inc., CA, United States) and synthesized by Sangon Biotechnology (Shanghai, China) and shown in Table 2. Quantitative RT-PCR analysis was performed on ABI 7,500 (Applied Biosystems). The cDNA was amplified in a 20 µL PCR reaction system containing 0.2 µM of each specific primer (Sangon, China) and of the SYBR Green master mix (TaKaRa Biotechnology) according to the manufacturer’s instructions. GAPDH was used as a housekeeping gene, and the gene expression levels were determined using the comparative 2^−∆∆CT^ method (Livak and Schmittgen, 2001).

**TABLE 2:** Primers used for real-time quantitative PCR.

Gene name	Genbank number	Primers position	PCR primers sequences (5′→3′)
*ADPN*	NM_206991	Forward	ACC​CAG​ACA​CAG​ATG​ACC​GTT
		Reverse	GAG​CAA​GAG​CAG​AGG​TAG​GAG​T
*AgRP*	XM_025154207.2	Forward	GGAACCGCAGGCATTGTC
		Reverse	GTA​GCA​GAA​GGC​GTT​GAA​GAA
*CART*	XM_003643097.5	Forward	CCGCACTACGAGAAGAAG
		Reverse	AGG​CAC​TTG​AGA​AGA​AAG​G
*CYP17A1*	NM_001001901.3	Forward	GCT​GAA​GCG​ATG​CCT​GAA​GGT​C
		Reverse	GGC​TCA​AGA​GGG​CTG​TTG​TTC​TC
*CYP19A1*	XM_046924620.1	Forward	GCC​AGT​TGC​CAC​AGT​GCC​TAT​C
		Reverse	GGC​CCA​ATT​CCC​ATG​CAG​TAT​CC
*ESR1*	NM_205183	Forward	TAT​TGA​TGA​TCG​GCT​TAG​TCT​GGG
		Reverse	CGA​GCA​GCA​GTA​GCC​AGT​AGC​A
*FSHβ*	NM_204257	Forward	GCT​TCA​CAA​GGG​ATC​CGG​TA
		Reverse	TGA​AGG​AGC​AGT​AGG​ATG​GC
*FSHR*	NM_205079	Forward	CAC​CAA​TGC​CAC​AGA​ACT​GAG​AT
		Reverse	GCA​CCT​TAT​GGA​CGA​CGG​GT
*GAPDH*	NM_204305	Forward	CTA​CAC​ACG​GAC​ACT​TCA​AG
		Reverse	ACA​AAC​ATG​GGG​GCA​TCA​G
*GH*	NM_204359	Forward	CTG​GAA​GAA​GGG​ATC​CAA​GCC
		Reverse	TAG​GTG​GGT​CTG​AGG​AGC​TG
*GHR*	NM_001001293	Forward	AGG​CTC​CTG​AGT​GAC​GAT​CAT​CTG
		Reverse	GCT​TGC​ACT​GAA​GTC​TGT​CTC​TGG
*Ghrelin*	NM_001001131	Forward	CCT​TGG​GAC​AGA​AAC​TGC​TC
		Reverse	CAC​CAA​TTT​CAA​AAG​GAA​CG
*GHRH*	XM_015296360	Forward	AGT​CAC​AAG​CTC​CAT​CTC​CTC​TCC
		Reverse	CTG​GGC​TGC​TCT​CAC​TGT​TTC​TG
*GnIH*	NM_204363	Forward	GCC​GAG​TGC​TTA​TTT​GCC​TTT​GAG
		Reverse	TCA​CAT​CCC​TGG​TTC​AGA​CTC​CTG
*GnIHR*	NM_204362.1	Forward	AGT​GGC​CTG​GTA​CAG​GGC​ATG​TCT
		Reverse	CAA​TGC​GGG​CAT​ACA​TGA​CGA​CAA
*GnRH1*	NM_001080877	Forward	TGG​GTT​TGT​TGA​TGG​TGT​TGT
		Reverse	ATT​TTC​CAG​CGG​GAA​GAG​TTG
*GnRH1R*	NM_204653	Forward	ACGGAGGGGGACACCAAC
		Reverse	GCC​CAG​CAC​TGC​TGT​ATT​GC
*IGF1R*	NM_205032	Forward	TTC​AGG​AAC​CAA​AGG​GCG​A
		Reverse	TGT​AAT​CTG​GAG​GGC​GAT​ACC
*LEPR*	NM_204323	Forward	TTT​GCT​GTT​GGG​CTT​TCT​TCA​C
		Reverse	AAC​CAG​ACC​GGC​TCC​GTA​CA
*LHβ*	XM_025153997	Forward	GTG​ACA​GTG​GCG​GTG​GAG​AA
		Reverse	CCCAAAGGGCTGCGGTA
*LHR*	AB009283	Forward	AGC​CTT​CCT​GCT​TTG​TCT​G
		Reverse	ATC​GTT​GTG​TAT​CCG​CCT​G
*NPY*	NM_205473.1	Forward	TGC​TGA​CTT​TCG​CCT​TGT​CG
		Reverse	GTG​ATG​AGG​TTG​ATG​TAG​TGC​C
*OVR*	NM_205229	Forward	ACT​GTG​AGG​ATG​GGT​CTG​ACG​A
		Reverse	TGG​GAT​ACA​CTG​GGT​TGA​CTG​AG
*POMC*	XM_015285103.3	Forward	CGCTACGGCGGCTTCA
		Reverse	TCT​TGT​AGG​CGC​TTT​TGA​CGA​T
*SIRT1*	XM_015288090	Forward	CGG​AAA​CAA​TAC​CTC​CAC​CTG​A
		Reverse	GAA​GTC​TAC​AGC​AAG​GCG​AGC​A

### Bone Quality Analysis

The excised bones were subjected to densitometry using the dual-energy x-ray absorptiometry (DXA) method on InAlyzer (Medikors LAB., Seoul, Korea). After the DXA analysis, bones were subjected to mechanical test analysis using a three-point method according to the method described by Song et al. (2020). Briefly, the bones were thawed to room temperature before the mechanical testing. The bone was rested in the anterior-posterior plane on two support points with 40 mm distance between the two support points (4 cm span) with a 1 N preload before loading to failure at a rate of 2 mm/min. The test was carried out by a microcomputer-controlled electronic universal testing machine (Jinan Shi JinNeng). Bone strength was calculated according to the formula “aw = (8*F*L)/π/d^3^, where F is the maximum force needed for fracture, L is the spacing between two support points, and d is the diameter of the bone.

After the bone-bending strength analysis, the bone volume was determined based on a modified procedure described by Cui et al. (2019). Briefly, the bone was inserted into a 100 ml measuring cylinder previously filled with some water. The bone volume was calculated by subtracting the initial volume from the final volume. Subsequently, the bones were degreased by immersing in 2:1 ethanol: benzene mixture for 96 h, air-dried under the fume hood for 24 h, then oven-dried at 105°C for 24 h and weighed to obtain the defatted dry weight. Subsequently, dry-defatted bones were placed in a muffle furnace at 550°C for ash content determination. The percentage of ash was calculated and expressed as a percentage of the dry-defatted bone weight. Bone ash concentration was calculated by dividing the ash weight by the volume of each bone.

### Statistical Analysis

Statistical analysis was performed using SAS software. For the production performance, egg quality, serum hormones and biochemical parameters, body weight, carcass weight, ovarian parameters and bone parameters, a two-way ANOVA model (version 8e; SAS Institute, Cary, NC) was used to estimate the main effect of energy and protein as well as their interaction. Results were expressed as means and standard errors. *p* < 0.05 was considered statistically significant.

## Results

### Laying Performance

The egg production, egg weight, egg mass, and feed intake did not differ among the dietary treatments (*p* > 0.05, Table 3).

**TABLE 3:** Effect of dietary metabolizable energy (E) and crude protein (P) levels during the prelay period (15–20 weeks of age) on production performance in laying hens at 72 weeks.

Parameters	Energy^a^	Protein^a^			SEM	Factor	*p*-Value
		15%	16.5%	Mean			
Laying rate, %^b^	2,700 kcal/kg	91.9	92.6	92.3	0.45	E	0.938
	2,800 kcal/kg	92.6	92.0	92.3	0.45	P	0.912
	Mean	92.3	92.3		0.63	E×P	0.326
Egg weight, g	2,700 kcal/kg	63.1	63.1	63.1	0.11	E	0.284
	2,800 kcal/kg	63.4	63.1	63.3	0.11	P	0.419
	Mean	63.3	63.1		0.16	E×P	0.329
Egg mass, g/d	2,700 kcal/kg	57.7	58.0	57.9	0.42	E	0.852
	2,800 kcal/kg	58.3	57.8	58.1	0.42	P	0.708
	Mean	58.0	57.9		0.59	E×P	0.510
Feed intake, g/d^b^	2,700 kcal/kg	118.8	117.8	118.3	1.57	E	0.900
	2,800 kcal/kg	121.0	116.7	118.9	1.57	P	0.316
	Mean	119.9	117.3		2.22	E×P	0.559

a Treatments were applied during prelay stage (15–20 wk of age). The same layer diet was fed after 20 wk of age.

b Total egg production from 24 to 72 weeks of age (*n* = 4).

### Egg Quality

Compared with the 15% CP group, the egg shape index and eggshell thickness were significantly higher in the 16.5% group (*p* < 0.05, Table 4). The yolk color was not affected by dietary protein level (*p* > 0.05), but decreased with a low-energy diet compared with a high-energy diet (*p* < 0.05). However, dietary energy and protein level did not affect the eggshell breaking strength, the albumen height, and Haugh units (*p* > 0.05).

**TABLE 4:** Effect of dietary metabolizable energy (E) and crude protein (P) levels during the prelay period (15–20 weeks of age) on egg quality of laying hens at 72 wk of age.

Parameters	Energy^a^	Protein^a^			SEM	Factor	*p*-Value
		15%	16.5%	Mean			
Egg shape index	2,700 kcal/kg	1.33	1.31	1.32	0.004	E	0.861
	2,800 kcal/kg	1.32	1.32	1.32	0.004	P	0.024
	Mean	1.33^a^	1.31^b^		0.006	E×P	0.195
Eggshell thickness, mm	2,700 kcal/kg	0.32	0.34	0.33	0.003	E	0.909
	2,800 kcal/kg	0.33	0.33	0.33	0.003	P	0.037
	Mean	0.32^a^	0.33^b^		0.004	E×P	0.116
Eggshell breaking strength, kg·f	2,700 kcal/kg	3.14	3.62	3.38	0.10	E	0.310
	2,800 kcal/kg	3.24	3.22	3.23	0.10	P	0.129
	Mean	3.19	3.42		0.15	E×P	0.098
Albumen height, mm	2,700 kcal/kg	6.57	6.62	6.59	0.09	E	0.414
	2,800 kcal/kg	6.33	6.66	6.49	0.09	P	0.133
	Mean	6.45	6.63		0.12	E×P	0.262
Yolk color	2,700 kcal/kg	6.13^a^	6.07^b^	6.12	0.13	E	0.043
	2,800 kcal/kg	6.55	6.43	6.49	0.13	P	0.615
	Mean	6.35	6.25		0.19	E×P	0.909
Haugh units	2,700 kcal/kg	77.79	78.35	78.07	0.67	E	0.327
	2,800 kcal/kg	76.17	78.10	77.14	0.67	P	0.193
	Mean	76.98	78.22		0.95	E×P	0.470

a Treatments were applied during prelay stage (15–20 week of age). The same layer diet was fed after 20 week of age.

^a,b^ Means within a row with different letters are significantly different at *p* < 0.05.

### Body Weight and Carcass Weight and Ovarian Parameters

BW, relative organ and muscle weight: BM, LM, and ovarian parameters did not differ among treatments (*p* > 0.05, Table 5, 6).

**TABLE 5:** Effect of dietary metabolizable energy (E) and crude protein (P) levels during the prelay period (15–20 weeks of age) on body weight, relative muscle and organ weight of laying hens at 72 wk of age.

Parameters	Energy^a^	Protein^a^			SEM	Factor	*p*-Value
		15%	16.5%	Mean			
BW, kg^b^	2,700 kcal/kg	2.16	2.03	2.15	0.04	E	0.296
	2,800 kcal/kg	2.16	2.14	2.09	0.04	P	0.146
	Mean	2.16	2.08		0.05	E×P	0.348
BM, %^c^	2,700 kcal/kg	10.97	11.16	11.06	0.28	E	0.414
	2,800 kcal/kg	10.61	10.84	10.73	0.28	P	0.610
	Mean	10.79	10.99		0.39	E×P	0.958
						
LM, %	2,700 kcal/kg	7.01	6.75	6.86	0.07	E	0.598
	2,800 kcal/kg	6.91	6.96	6.94	0.07	P	0.519
	Mean	6.96	6.85		0.10	E×P	0.174
Liver weight, %	2,700 kcal/kg	1.87	2.03	1.95	0.07	E	0.884
	2,800 kcal/kg	1.87	2.08	1.97	0.07	P	0.119
	Mean	1.87	2.05		0.11	E×P	0.771
Abdominal fat weight, %	2,700 kcal/kg	5.67	5.18	5.43	0.40	E	0.631
	2,800 kcal/kg	5.91	5.50	5.71	0.40	P	0.444
	Mean	5.79	5.34		0.57	E×P	0.945
Ovary weight, %	2,700 kcal/kg	0.014	0.013	0.013	0.001	E	0.957
	2,800 kcal/kg	0.014	0.013	0.013	0.001	P	0.385
	Mean	0.014	0.013		0.001	E×P	0.926
						
Oviduct, %	2,700 kcal/kg	4.12	4.21	4.17	0.26	E	0.926
	2,800 kcal/kg	3.65	4.61	4.13	0.26	P	0.182
	Mean	3.89	4.41		0.37	E×P	0.259
Magnum, g	2,700 kcal/kg	63.80	58.27	61.09	6.18	E	0.744
	2,800 kcal/kg	51.44	64.78	58.11	6.18	P	0.663
	Mean	57.62	61.52		8.74	E×P	0.302
Eggshell gland, g	2,700 kcal/kg	22.89	23.65	23.27	1.77	E	0.319
	2,800 kcal/kg	23.87	27.86	25.87	1.77	P	0.361
	Mean	23.38	25.76		2.50	E×P	0.530

a Treatments were applied during prelay stage (15–20 week of age). The same layer diet was fed after 20 week of age.

b BW, at 72week of age; BW, body weight; BM, breast muscle; LM, leg muscle; (*n* = 4).

c Muscle and Organ weights expressed as % live weight.

**TABLE 6:** Effect of dietary metabolizable energy (E) and crude protein (P) levels during the prelay period (15–20 weeks of age) on ovarian parameters of laying hens at 72 week of age.

Parameters	Energy^a^	Protein^a^			SEM	Factor	*p*-Value
		15%	16.5%	Mean			
Number of dominant follicles	2,700 kcal/kg	5.63	5.50	5.56	0.29	E	1.000
	2,800 kcal/kg	5.75	5.38	5.56	0.29	P	0.555
	Mean	5.69	5.44		0.41	E×P	0.767
Weight of dominant follicles, g	2,700 kcal/kg	38.40	37.93	38.16	2.78	E	0.417
	2,800 kcal/kg	41.13	41.83	41.48	2.78	P	0.978
	Mean	39.76	39.88		3.94	E×P	0.884
Number of small yellow follicles	2,700 kcal/kg	18.25	17.00	17.63	1.04	E	0.309
	2,800 kcal/kg	19.38	19.00	19.19	1.04	P	0.591
	Mean	18.81	18.00		1.47	E×P	0.771
Weight of small yellow follicles, g	2,700 kcal/kg	2.95	2.65	2.80	0.28	E	0.550
	2,800 kcal/kg	3.20	2.89	3.04	0.28	P	0.455
	Mean	3.08	2.77		0.40	E×P	0.988
Number of large white follicles	2,700 kcal/kg	28.25	22.88	25.56	1.30	E	0.510
	2,800 kcal/kg	25.63	23.00	24.31	1.30	P	0.051
	Mean	26.94	22.94		1.84	E×P	0.469
Weight of large white follicles, g	2,700 kcal/kg	0.84	1.01	0.92	0.12	E	0.383
	2,800 kcal/kg	0.80	0.73	0.77	0.12	P	0.762
	Mean	0.82	0.89		0.18	E×P	0.503

a Treatments were applied during prelay stage (15–20 week of age). The same layer diet was fed after 20 week of age.

### Serum Parameters in Laying Hens

There was no energy and protein level and their interaction effect on serum Ca concentration (*p* > 0.05, Table 7). Compared with the 15% CP group, the serum P concentration was significantly higher in the 16.5% CP group (*p* < 0.05), and the interaction of energy/protein level showed a trend to influence on P concentration (*p* = 0.063). However, different levels of ME and CP and interaction had no significant effect on the concentration of serum TP, ALP, E_2_, FSH, LH, and P_4_ (*p* > 0.05, Table 7).

**TABLE 7:** Effect of dietary metabolizable energy (E) and crude protein (P) levels during the prelay period (15–20 weeks of age) on serum parameters in laying hens at 72 week of age.

Parameters	Energy^a^	Protein^a^			SEM	Factor	*p*-Value
		15%	16.5%	Mean			
Ca, mmol/L	2,700 kcal/kg	8.84	8.42	8.63	0.67	E	0.557
	2,800 kcal/kg	7.99	10.41	9.20	0.67	P	0.310
	Mean	8.41	9.41		0.92	E×P	0.158
P, mmol/L	2,700 kcal/kg	1.61	1.77	1.69	0.17	E	0.283
	2,800 kcal/kg	1.38	2.56	1.97	0.17	P	0.019
	Mean	1.50^b^	2.16^a^		0.25	E×P	0.063
TP, g/L	2,700 kcal/kg	53.49	53.38	53.43	2.35	E	0.824
	2,800 kcal/kg	49.24	56.11	52.68	2.35	P	0.328
	Mean	51.36	55.74		3.32	E×P	0.313
ALP, U/L	2,700 kcal/kg	762.5	978.8	870.6	264.4	E	0.469
	2,800 kcal/kg	504.0	677.5	590.8	264.4	P	0.612
	Mean	633.3	828.1		374.0	E×P	0.955
E_2_, Pg/mL	2,700 kcal/kg	309.28	404.13	356.70	33.10	E	0.455
	2,800 kcal/kg	299.35	330.85	315.10	33.10	P	0.264
	Mean	304.31	367.49		53.88	E×P	0.568
FSH, mIU/mL	2,700 kcal/kg	2.33	4.83	3.58	0.56	E	0.360
	2,800 kcal/kg	3.00	2.65	2.83	0.56	P	0.198
	Mean	2.66	3.74		0.79	E×P	0.098
LH, mIU/mL	2,700 kcal/kg	4.94	5.58	5.26	0.51	E	0.806
	2,800 kcal/kg	4.93	5.94	5.44	0.51	P	0.277
	Mean	4.93	5.76		0.72	E×P	0.805
P_4_, ng/mL	2,700 kcal/kg	1.36	0.75	1.06	0.21	E	0.789
	2,800 kcal/kg	1.15	0.81	0.98	0.21	P	0.135
	Mean	1.25	0.78		0.30	E×P	0.657

a Treatments were applied during prelay stage (15–20 week of age). The same layer diet was fed after 20 wk of age.

^a,b^ Means within a row with different letters are significantly different at *p* < 0.05.

Ca, calcium; P, phosphorus; TP, total protein; ALP, alkaline phosphatase; E_2_, estradiol; FSH, follicle-stimulating hormone; LH, luteinizing hormone; P_4_, progesterone (*n* = 4).

### The mRNA Expression of Genes in the Hypothalamus-Pituitary-Ovarian Axis

**FIGURE 1:**
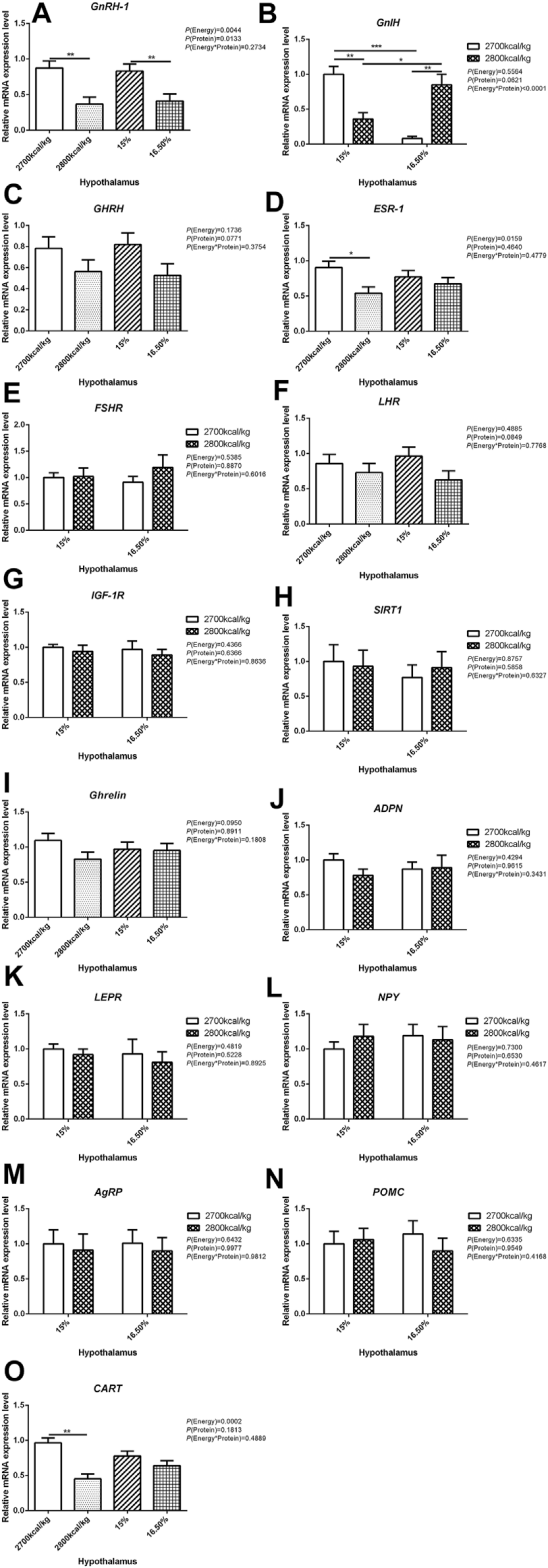
Effect of dietary metabolizable energy (E) and crude protein (P) levels during the prelay period (15–20 weeks of age) on the mRNA expression of genes *GnRH-1* **(A)**, *GnIH* **(B)**, *GHRH* **(C)**, *ESR-1* **(D)**, *FSHR* **(E)**, *LHR* **(F)**, *IGF-1R* **(G)**, *SIRT1* **(H)**, *Ghrelin* **(I)**, *ADPN* **(J)**, *LEPR* **(K)**, *NPY* **(L)**, *AgRP* **(M)**, *POMC* **(N)**, and *CART* **(O)** in the hypothalamus of hens. Data represent the mean ± SD (*n* = 4); ∗*p* < 0.05, ∗∗*p* < 0.01, ∗∗∗*p* < 0.0001.

The mRNA expression level of *GnRH-1*, *ESR-1*, and *CART* in the hypothalamus decreased when the dietary energy level increased from 2,700 kcal/kg to 2,800 kcal/kg (*p* < 0.05; Figures 1A,D,O) while the mRNA expression level of *GnRH-1* and *LHR* in the hypothalamus decreased when dietary protein level increased from 15% to 16.5% (*p* < 0.05; Figure 1A). However, the interaction between dietary energy and protein levels only affected the mRNA expression level of *GnIH*. When the protein level of diet was 15%, the mRNA expression of *GnIH* decreased with the increase in dietary energy level, while the protein level was 16.5%, the expression of *GnIH* increased (*p* < 0.05; Figure 1B). The mRNA expression level of *GHRH*, *FSHR*, *IGF-1R*, *SIRT1*, *Ghrelin*, *ADPN*, *LEPR*, *NPY*, *AgRP*, and *POMC* in the hypothalamus was not influenced by the dietary energy and protein levels and their interaction (Figures 1C,E,G–N).

**FIGURE 2:**
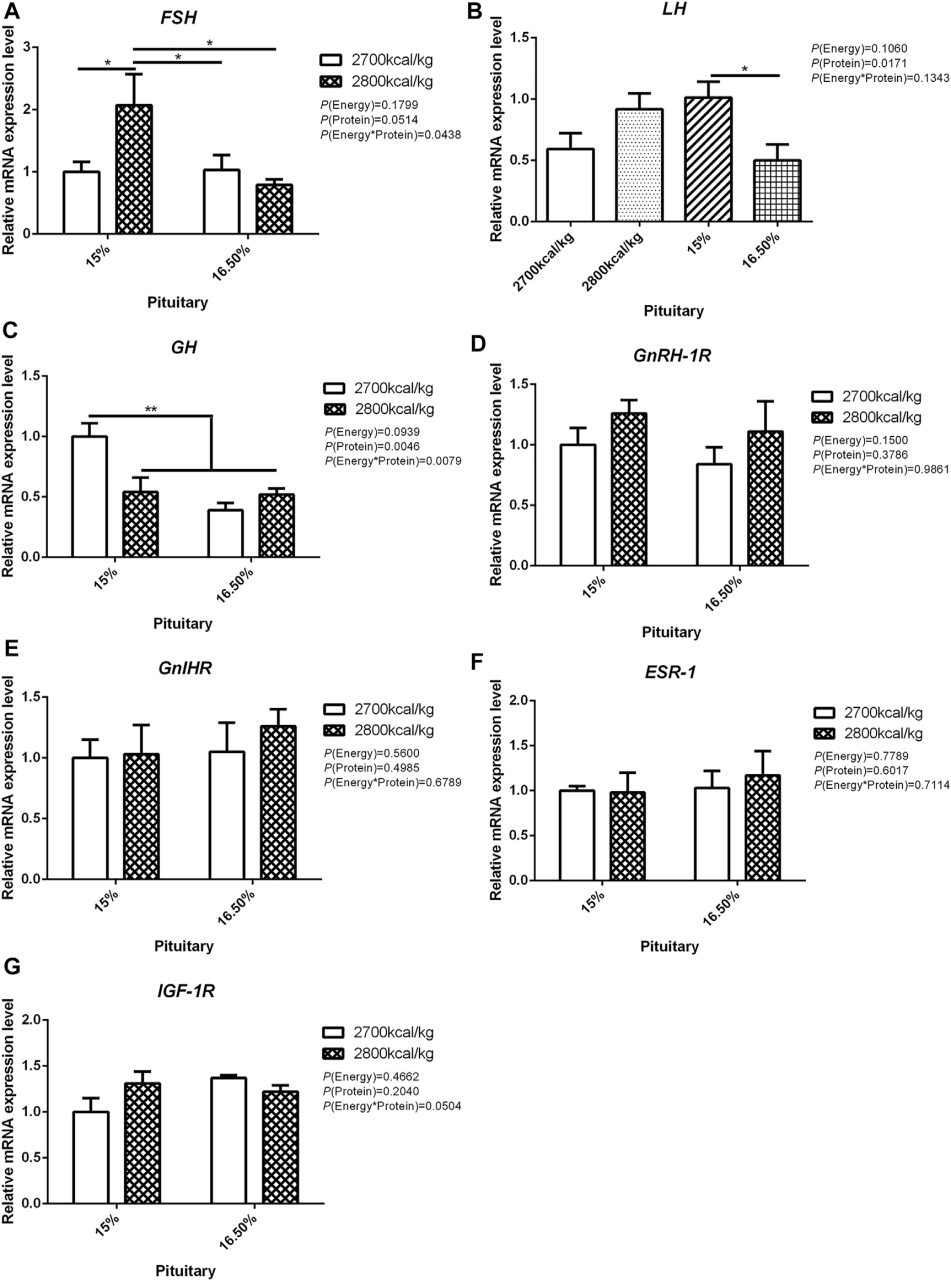
Effect of dietary metabolizable energy (E) and crude protein (P) levels during the prelay period (15–20 weeks of age) on the mRNA expression of genes *FSH* **(A)**, *LH* **(B)**, *GH* **(C)**, *GnRH-1R* **(D)**, *GnIHR* **(E)**, *ESR-1* **(F)**, and *IGF-1R* **(G)** in the pituitary of hens. Data represent the mean ± SD (*n* = 4); ∗*p* < 0.05, ∗∗*p* < 0.01.

In the pituitary, the mRNA expression level of *FSH* and *GH* were affected by the interaction between the dietary energy and protein level (*p* < 0.05; Figures 2A,C). When the protein level of diet was 15%, the mRNA expression of *FSH* increased with the increase in dietary energy level, while the mRNA expression of *GH* decreased (*p* < 0.05; Figures 2A,C). The expression of LH was decreased by dietary protein (*p* < 0.05) but not by energy and the interaction of energy and protein level (*p* > 0.05, Figure 2B). The mRNA expression level of *GnRH-1R*, *GnIHR*, *ESR-1*, and *IGF-1R* in the pituitary was not influenced by the dietary energy and protein levels and their interaction (Figures 2D–G).

**FIGURE 3:**
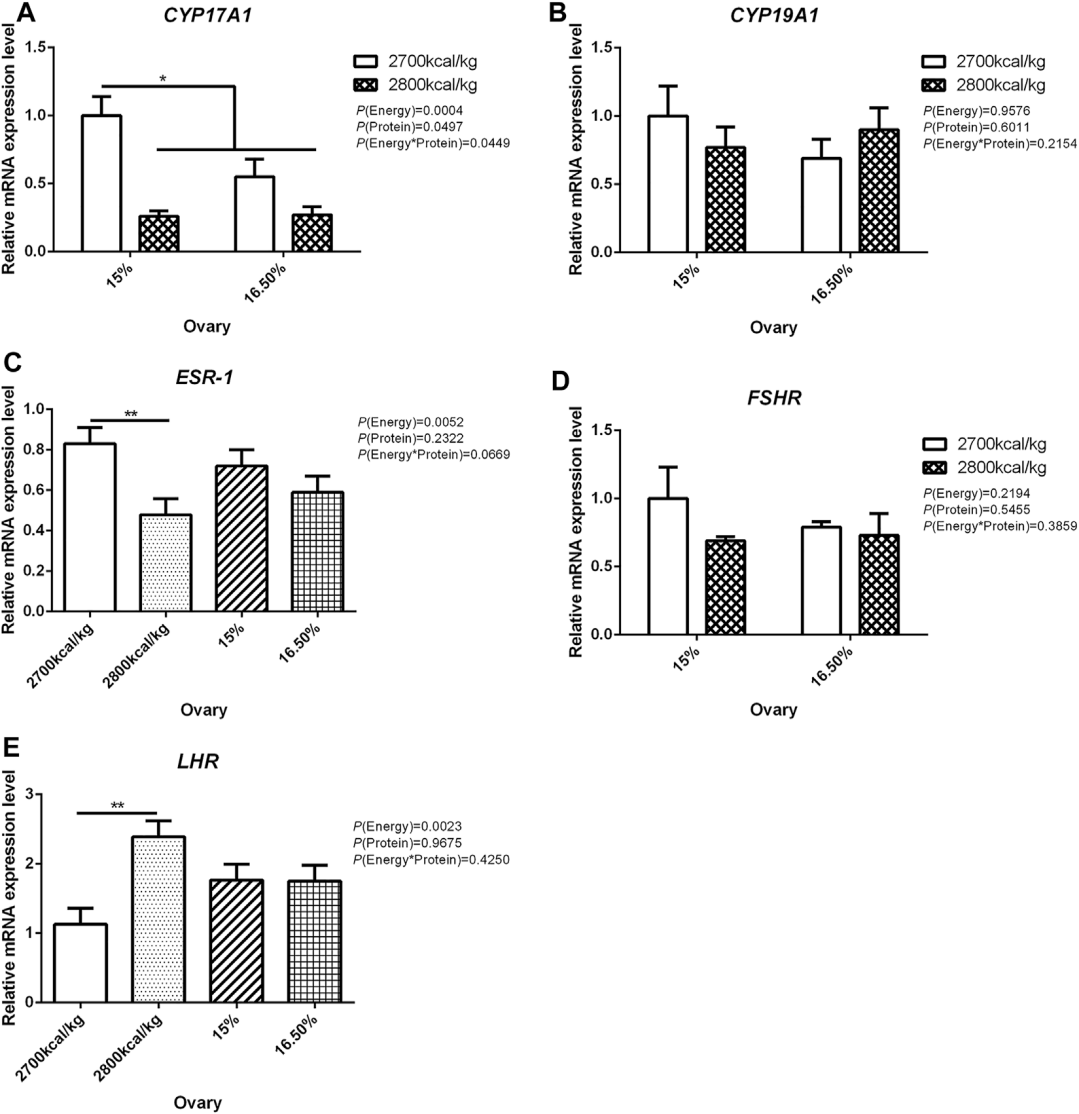
Effect of dietary metabolizable energy (E) and crude protein (P) levels during the prelay period (15–20 weeks of age) on the mRNA expression of genes *CYP17A1* **(A)**, *CYP19A1* **(B)**, *ESR-1* **(C)**, *FSHR* **(D)**, and *LHR* **(E)** in the ovary of hens. Data represent the mean ± SD (*n* = 4); ∗*p* < 0.05, ∗∗*p* < 0.01.

In the ovary, the mRNA expression level of *CYP17A1* was affected by the interaction between the dietary energy and protein level (*p* < 0.05; Figure 3A). When the protein level of diet was 15%, the mRNA expression of *CYP17A1* decreased with the increase in dietary energy level (*p* < 0.05; Figure 3A). In the ovary, compared with hens fed with 2,700 kcal/kg diet, the mRNA expression level of *ESR-1* was significantly decreased by 2,800 kcal/kg diet (*p* < 0.05; Figure 3C). The mRNA expression level of *LHR* in the ovary increased when the dietary energy level increased from 2,700 kcal/kg to 2,800 kcal/kg (*p* < 0.05; Figure 3E). The mRNA expression level of *CYP19A1* and *FSHR* in the ovary was not influenced by dietary energy level, dietary protein level, and their interaction (Figures 3B,D).

### The mRNA Expression in the Follicles

**FIGURE 4:**
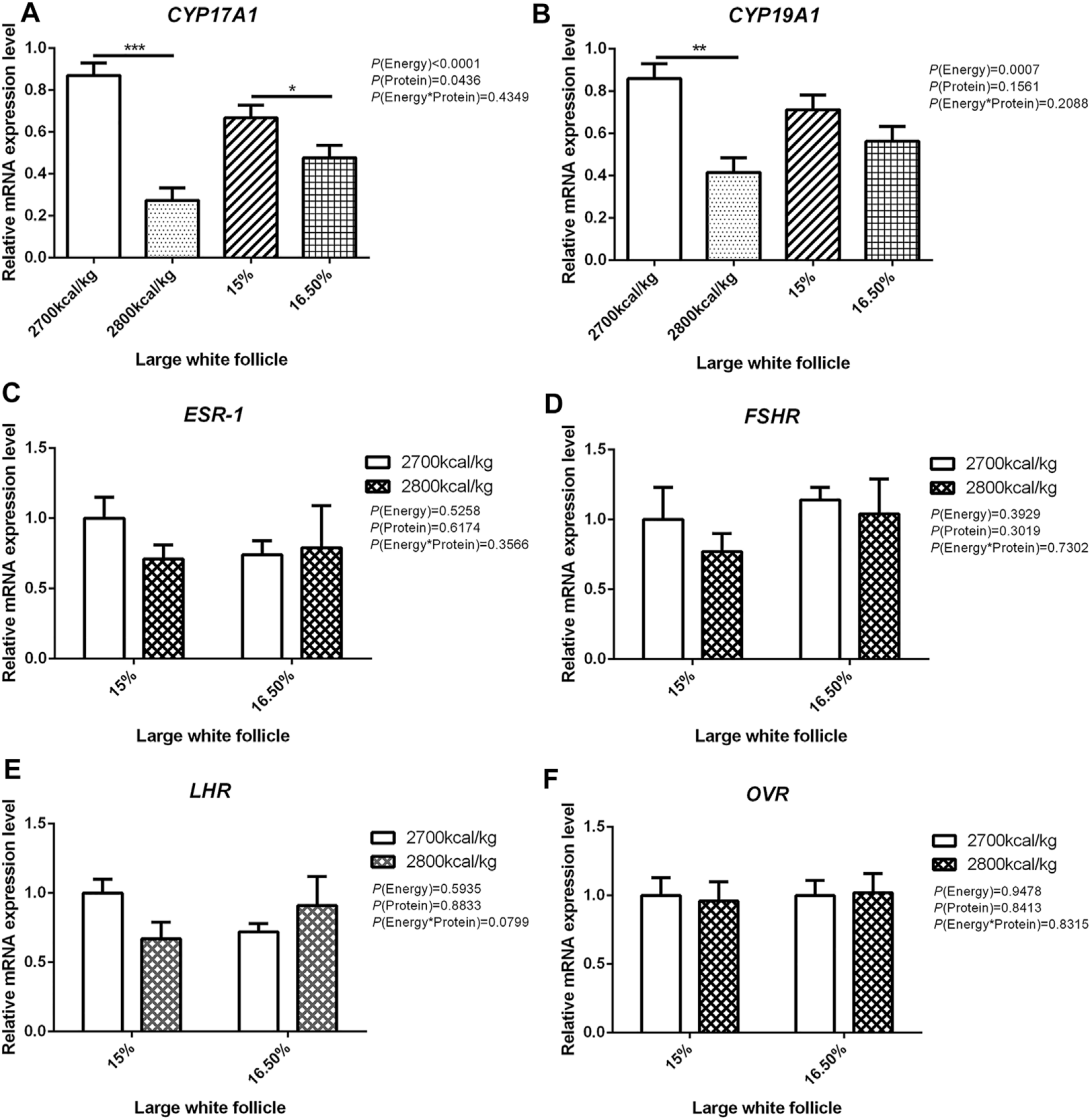
Effect of dietary metabolizable energy (E) and crude protein (P) levels during the prelay period (15–20 weeks of age) on the mRNA expression of genes *CYP17A1* **(A)**, *CYP19A1* **(B)**, *ESR-1* **(C)**, *FSHR* **(D)**, *LHR* **(E)**, and *OVR* **(F)**in the large white follicles of hens. Data represent the mean ± SD (*n* = 4); ∗*p* < 0.05, ∗∗*p* < 0.01, ∗∗∗*p* < 0.0001.

**FIGURE 5:**
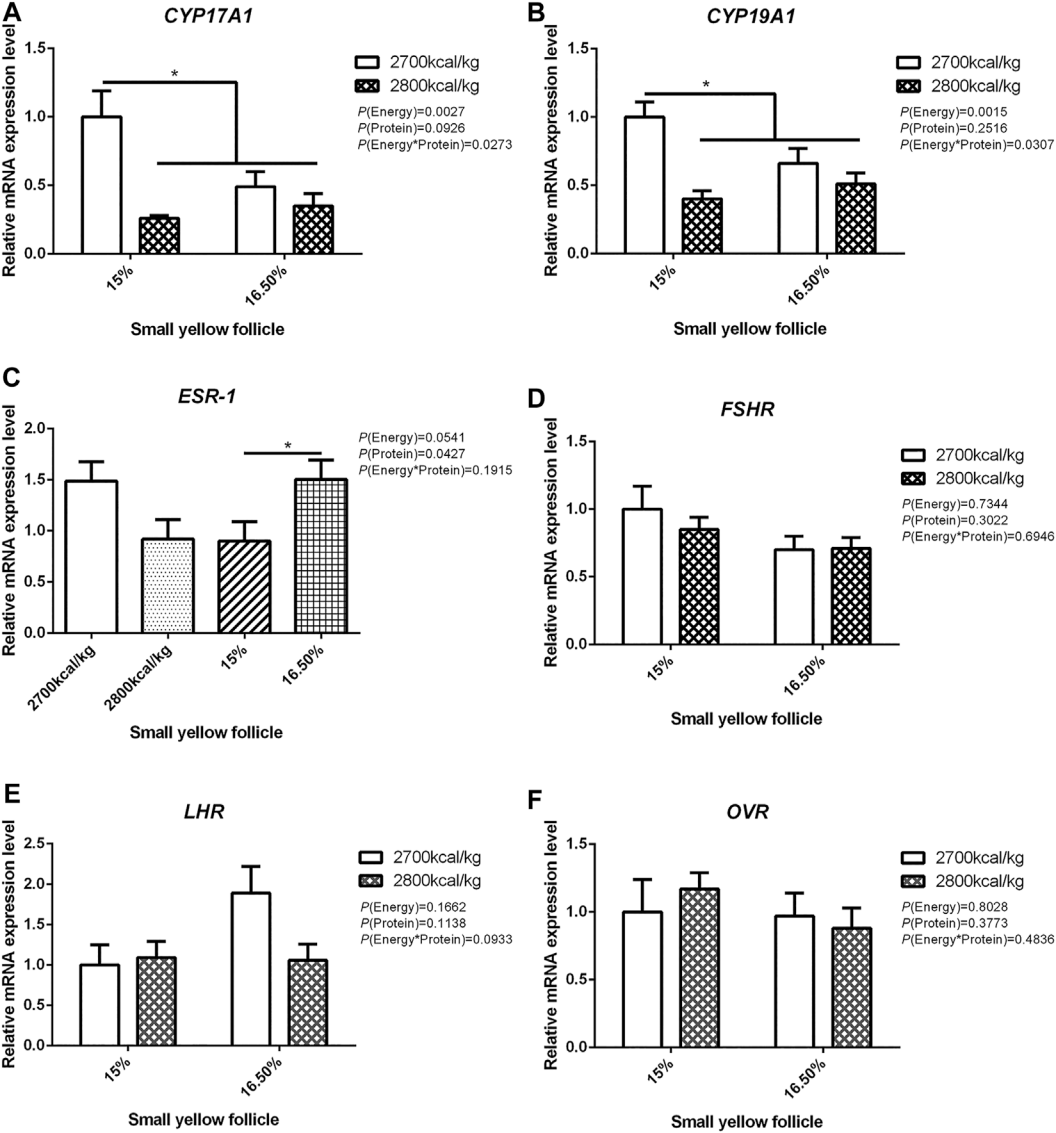
Effect of dietary metabolizable energy (E) and crude protein (P) levels during the prelay period (15–20 weeks of age) on the mRNA expression of genes *CYP17A1* **(A)**, *CYP19A1* **(B)**, *ESR-1* **(C)**, *FSHR* **(D)**, *LHR* **(E)**, and *OVR* **(F)** in the small yellow follicles of hens. Data represent the mean ± SD (*n* = 4); ∗*p* < 0.05.

**FIGURE 6:**
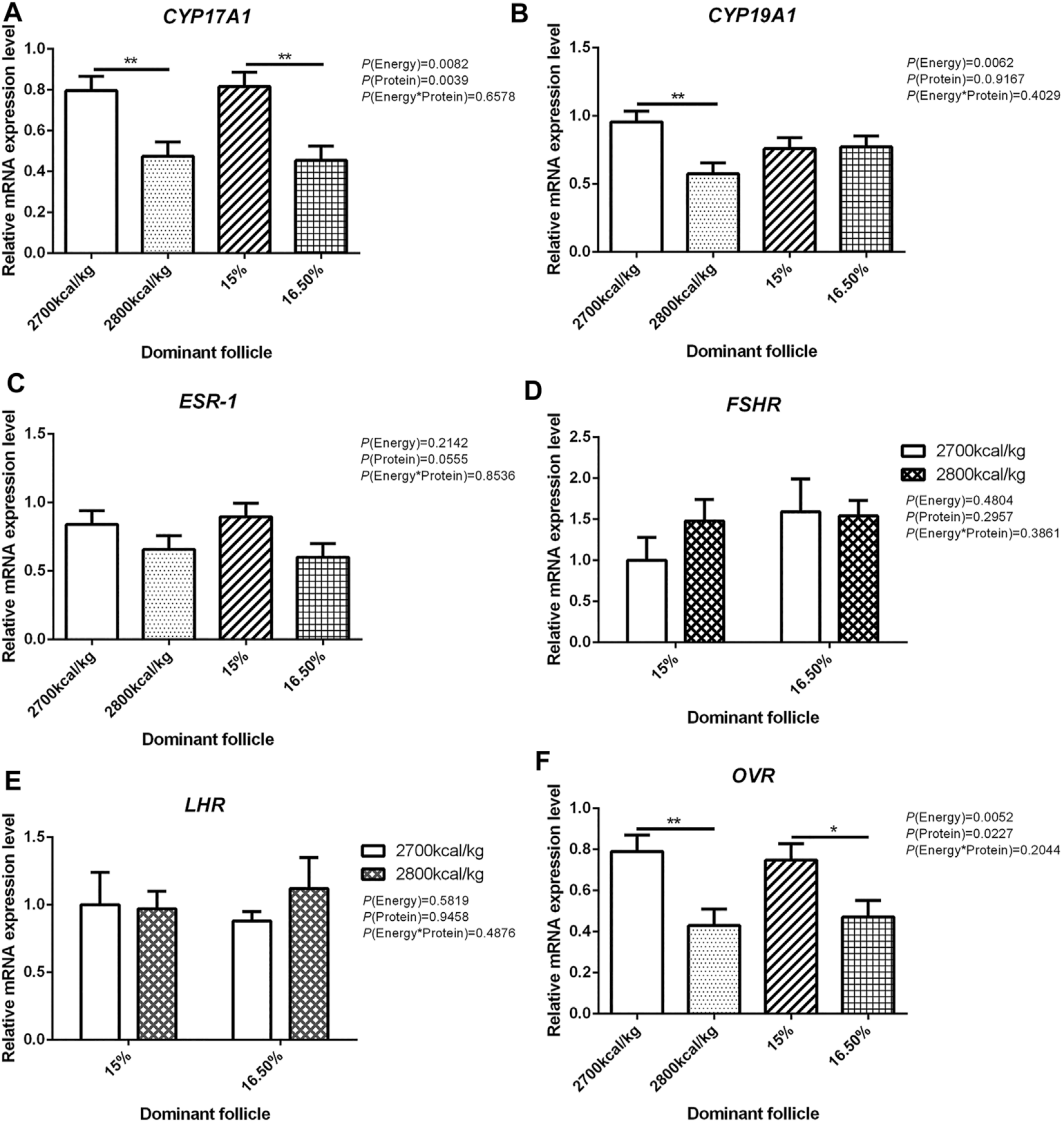
Effect of dietary metabolizable energy (E) and crude protein (P) levels during the prelay period (15–20 weeks of age) on the mRNA expression of genes *CYP17A1* **(A)**, *CYP19A1* **(B)**, *ESR-1* **(C)**, *FSHR* **(D)**, *LHR* **(E)**, and *OVR* **(F)** in the dominant follicles of hens. Data represent the mean ± SD (*n* = 4); ∗*p* < 0.05, ∗∗*p* < 0.01.

The mRNA expression level of *CYP17A1* and *CYP19A1* in the large white follicles decreased when dietary energy level increased from 2,700 kcal/kg to 2,800 kcal/kg (*p* < 0.05; Figures 4A,B), and the mRNA expression level of *CYP19A1* was decreased then dietary protein level increased from 15 to 16.50% (*p* < 0.05, Figure 4B). The mRNA expression level of *ESR-1*, *FSHR*, *LHR*, and *OVR* in the large white follicles was not influenced by the dietary energy and protein level and their interaction (Figures 4C–F). In the small yellow follicles, the mRNA expression level of *CYP17A1* and *CYP19A1* were affected by the interaction between the dietary energy and protein level (*p* < 0.05; Figures 5A,B). When the protein level of diet was 15%, the mRNA expression of *CYP17A1* and *CYP19A1* decreased with the increase in dietary energy level (*p* < 0.05; Figures 5A,B). In the small yellow follicles, the mRNA expression level of *ESR-1* tended to be decreased when the dietary energy level increased from 2,700 kcal/kg to 2,800 kcal/kg (*p* = 0.054)and increased when the dietary protein level increased from 15 to 16.5% (*p* < 0.05; Figure 5C). The mRNA expression level of *FSHR*, *LHR* and *OVR* in the small yellow follicles was not influenced by the dietary energy and protein levels, and their interaction (Figures 5D–F). In the dominant follicles, the mRNA expression level of *CYP17A1*, *CYP19A1*, and *OVR* decreased when the dietary energy level increased from 2,700 kcal/kg to 2,800 kcal/kg (*p* < 0.05; Figures 6A,B,F). When the dietary protein level increased from 15 to 16.5%, the mRNA expression level of *ESR-1* show a trend to decrease (*p* = 0.055; Figure 6C) whereas *CYP17A1* and *OVR* decreased (*p* < 0.05; Figures 6A,F). The mRNA expression level of *FSHR* and *LHR* in the dominant follicles was not influenced by the dietary energy and protein level, and their interaction (Figures 6D,E).

### Bone Parameters

The effects of energy and protein levels on bone physical characteristics, bone mineral density (BMD), and bone-bending strength (BBS) are shown in Table 8. At 72 wk, the length and width of bone, relative bone weight, BMD, and BBS of the tibia were not affected by energy or protein level (*p* > 0.05), nor was there any interaction effect (*p* > 0.05).

**TABLE 8:** Effect of dietary metabolizable energy (E) and crude protein (P) levels during the prelay period (15–20 weeks of age) on bone parameters in laying hens at 72 week of age.

Parameters	Energy^a^	Protein^a^			SEM	Factor	*p*-Value
		15%	16.5%	Mean			
Femur							
Length, mm	2,700 kcal/kg	70.3	69.9	70.06	5.46	E	0.168
	2,800 kcal/kg	82.3	80.5	81.38	5.46	P	0.893
	Mean	76.25	75.19		7.72	E×P	0.931
Width, mm	2,700 kcal/kg	6.66	7.56	7.11	0.37	E	0.272
	2,800 kcal/kg	7.66	7.77	7.71	0.37	P	0.356
	Mean	7.16	7.66		0.52	E×P	0.470
Weight, %	2,700 kcal/kg	0.44	0.45	0.45	0.01	E	0.646
	2,800 kcal/kg	0.43	0.44	0.43	0.01	P	0.763
	Mean	0.44	0.44		0.02	E×P	0.711
BMD, g/cm2	2,700 kcal/kg	0.31	0.31	0.33	0.01	E	0.213
	2,800 kcal/kg	0.32	0.34	0.31	0.01	P	0.631
	Mean	0.32	0.32		0.02	E×P	0.454
BBS, MPa	2,700 kcal/kg	0.04	0.04	0.04	0.003	E	0.994
	2,800 kcal/kg	0.04	0.04	0.04	0.003	P	0.853
	Mean	0.04	0.04		0.004	E×P	0.660
Tibia							
Length, mm	2,700 kcal/kg	117.4	114.4	115.9	4.87	E	0.531
	2,800 kcal/kg	120.6	102.3	111.4	4.87	P	0.147
	Mean	119.0	108.3		6.89	E×P	0.286
Width, mm	2,700 kcal/kg	6.54	6.36	6.45	0.14	E	0.808
	2,800 kcal/kg	6.62	6.39	6.50	0.14	P	0.325
	Mean	6.58	6.38		0.20	E×P	0.903
Weight, %	2,700 kcal/kg	0.51	0.51	0.51	0.01	E	0.164
	2,800 kcal/kg	0.52	0.54	0.53	0.01	P	0.462
	Mean	0.52	0.53		0.02	E×P	0.507
BMD, g/cm2	2,700 kcal/kg	0.29	0.26	0.28	0.01	E	0.216
	2,800 kcal/kg	0.28	0.31	0.29	0.01	P	0.955
	Mean	0.28	0.29		0.02	E×P	0.108
BBS, MPa	2,700 kcal/kg	0.06	0.06	0.06	0.009	E	0.451
	2,800 kcal/kg	0.07	0.08	0.07	0.009	P	0.678
	Mean	0.06	0.07		0.01	E×P	0.550

a Treatments were applied during prelay stage (15–20 week of age). The same layer diet was fed after 20 week of age.

BMD, bone mineral density; BBS, bone bending strength, *n* = 4.

### Ash Content

The effects of energy and protein levels on ash weight, ash concentration, and ash percentage of femur and tibia are shown in Table 9. At 72 wk of age, there was no effect of energy or protein level on the ash weight and ash concentration of femur and tibia (*p* > 0.05). There was an energy/protein interaction on both femur and tibial ash weight (*p* < 0.05); the hen fed with the 2,800 ME-16.5%CP diet or 2,700 ME-15%CP diet had a higher ash weight (*p* < 0.05).

**TABLE 9:** Effect of dietary metabolizable energy (E) and crude protein (P) levels during the prelay period (15–20 weeks of age) on bone ash in laying hens at 72 week of age.

Parameters	Energy^a^	Protein^a^			SEM	Factor	*p*-Value
		15%	16.5%	Mean			
Femur							
Ash weight, g	2,700 kcal/kg	2.62^x^	2.20^y^	2.41	0.07	E	0.714
	2,800 kcal/kg	2.38^xy^	2.52^x^	2.45	0.07	P	0.204
	Mean	2.50	2.36		0.10	E×P	0.019
Ash concentration, g/cm^3^	2,700 kcal/kg	0.36	0.31	0.33	0.02	E	0.708
	2,800 kcal/kg	0.34	0.35	0.34	0.02	P	0.480
	Mean	0.35	0.33		0.03	E×P	0.236
Ash, %	2,700 kcal/kg	46.95	44.26	45.61	1.90	E	0.983
	2,800 kcal/kg	45.96	45.38	45.67	1.90	P	0.555
	Mean	46.45	44.82		2.69	E×P	0.702
Tibia							
Ash weight, g	2,700 kcal/kg	4.12^x^	3.68^y^	3.65	0.16	E	0.606
	2,800 kcal/kg	3.68^xy^	3.86^x^	3.77	0.16	P	0.129
	Mean	3.90	3.52		0.23	E×P	0.032
Ash concentration, g/cm^3^	2,700 kcal/kg	0.44	0.38	0.41	0.01	E	0.549
	2,800 kcal/kg	0.40	0.40	0.40	0.01	P	0.097
	Mean	0.42	0.39		0.02	E×P	0.118
Ash, %	2,700 kcal/kg	52.19	51.03	51.01	1.15	E	0.655
	2,800 kcal/kg	51.03	49.49	50.26	1.15	P	0.252
	Mean	51.61	49.66		1.62	E×P	0.801

a Treatments were applied during prelay stage (15–20 week of age). The same layer diet was fed after 20 week of age.

^x,y^ Mean within interaction with different letters are significantly different at *p* < 0.05, *n* = 4.

## Discussion

In the current study, egg production was not influenced by dietary energy and protein levels during the prelay period, and all dietary treatment groups reached 50% production at similar ages (24 wk of age). In addition, egg weight and egg mass was similar among the treatment, neither influenced by the energy level nor protein level. These findings are in line with those found in the literature, where dietary protein levels prior to sexual maturity did not influence egg production (Hussein et al., 1996; Keshavarz, 1998). However, a previous study has shown a positive effect of prelay diet on production performance (Sujatha et al., 2014). They reported that an increase in energy and protein levels in a prelay diet improves production performance. The reason for the discrepancy between experimental results is not apparent but is likely to be due to the difference in the energy level. Sujatha et al. (2014) increased the energy level from 2,500 to 2,700 kcal/kg, whereas the dietary treatment in this experiment was 2,700 and 2,800 kcal/kg. This suggests that 2,700 kcal/kg energy meets the growth requirement, and further increase to 2,800 kcal does not affect the production performance. In addition, the difference in egg-type strains used for the study can be a reason for the discrepancy in the result. Hy-Line brown pullets were used in our study, whereas White Leghorn pullets were used for their study.

Earlier studies reported that increasing the energy level of the diet from 2,850 kcal/kg to 3,050 kcal/kg did not influence the eggshell quality and Haugh units (Junqueira et al., 2006). This is consistent with the result of the current study. Yolk color has a considerable influence on the marketability of eggs; the color of the yolk depends on the fat-soluble carotenoids and xanthophylls (Gunawardana et al., 2008; Pérez-Bonilla et al., 2012). We observed that the yolk color increased with the increase of the dietary energy level. Similar findings were reported by Pérez-Bonilla et al. (2012), who found that yolk pigmentation increased linearly with an increase in energy concentration of the diet even though all diets had similar levels of pigmenting additives and corn. The BW and relative organ weight at the end-of-lay was similar among the dietary treatments suggesting that dietary energy and protein levels during the prelay period had no effect on the BW and relative organ weight at the end-of-lay. This observation corroborates those of Lilburn and Myers-Miller (1990), who found that protein intake during the prelay period (18–25 wk) did not influence BW and carcass parameters in broiler breeder pullets. Furthermore, Joseph et al. (2000) found that increased CP levels in the diet of prelay broiler breeders had no influence on BW and relative organ weight.

In poultry, ovulation and egg production are mainly regulated by the HPG axis, in which the endocrine system plays a dominant role. The hypothalamus is the main site of the regulatory axis. The regulation of energy homeostasis is inseparable from the central nervous system, especially the hypothalamus. In the present study, the endocrine system in the HPG axis was evaluated in aged hens around the end of the laying cycle. Sharp et al. (1992) found that LH in plasma and pituitary decreased and LH responsiveness to GnRH decreased in laying hens with low laying rate or no production. Earlier studies have demonstrated that dietary energy levels may promote the secretion of GnRH, FSH, LH, and E_2_ to promote follicular development and reproductive performance (Scaramuzzi et al., 2006; Romano et al., 2007; Zhou et al., 2010). Similarly, the plasma LH and FSH concentrations of broiler breeder pullets fed with high energy diet increased compared to the low energy diet (Hadinia et al., 2020). It was found that the decrease in egg production was related to the decrease of LH and FSH in the SYF and plasma (Sharp et al., 1992; Ciccone et al., 2005). Tanabe et al. (1981) and Wang and Johnson (1993) found that high LH level was associated with high laying rate in laying hens. However, our results showed that the dietary treatment did not significantly influence the serum concentration of E_2_ and LH of aged hens. This could explain the similarity in the production performance among the dietary treatment. It has been found that the decrease of plasma LH is related to the decrease of *GnRH-1* mRNA in the hypothalamus of aging layers with ovarian degeneration (Dunn et al., 1996). In our experiment, there was no change in the concentration of serum LH. The *GnRH-1* mRNA in the hypothalamus was the highest in hens fed with 2,700 kcal/kg diet or 15% CP diet. In the pituitary, the mRNA expression level of *LH* is up-regulated in the 15% CP diet and showed a trend to be increased by 2,700 kcal/kg diet. We think that the increase of GnRH-1 secretion in the hypothalamus leads to the increase of *LH* mRNA level in the pituitary.

The development of ovarian follicles is mainly regulated by FSH and LH secreted by the pituitary gland. It was found that FSH treatment alone could increase FSH binding capacity and *FSHR* mRNA level, promote follicular development, and ovulation in hypophysectomized rats pre-treated with estrogen (Galway et al., 1990). It has been reported that high dietary energy levels can promote *FSHR* and *LHR* gene mRNA expression in the ovary (Zhou et al., 2009). Our study found that the mRNA expression level of *LH* in the pituitary was the highest in the group with 15% CP whereas the expression of *LHR* in the ovary, LWF, SYF, and DF was not obvious altered, indicating that dietary protein play a minor role in affecting the expression of *LH*. In contrast, the mRNA level of *LHR* in the ovary was lower in 2,700 kcal/kg groups, suggesting that dietary energy is an important factor affecting the expression of *LH*. The mRNA level of *ESR-1* in the hypothalamus and ovary was highest in the 2,700 kcal/kg groups, which may be caused by positive feedback regulation of the HPG axis. Moreover, the increased *ESR-1* mRNA level by 16.5% CP diet in SYF was not observed in LWF or DF, suggesting that the response of ESR-1 is dependent of the stage of follicle development.

During the laying cycle, only one yolk follicle is selected as the dominant follicle and enters the stage of development, becoming the pre-ovulatory follicle, and then the follicles develop in the order of size until ovulation (Onagbesan et al., 2009). The more follicles selected to enter the development stage, the longer the laying cycle and the higher the laying performance of poultry. The decrease in egg production is related to the decrease of collection of ovarian eggs running to yolk follicles (Waddington et al., 1985; Palmer and Bahr, 1992). Previous studies have shown that feeding regimes can influence the development of ovarian follicles, and broiler breeder hens fed ad libitum had more pre-ovulatory ovarian follicles (Bacon et al., 1972; Hocking et al., 1987; Yu et al., 1992). However, there was no significant difference in the number and weight of follicles of different grades among the groups in this study. Similar findings were reported by Joseph et al. (2000), where CP level in prelay diet had no remarkable effect on ovarian parameters.

Estrogen is mainly secreted by theca cells and granulosa cells of the ovary, which regulates the development of follicle, reproductive tract, and ovulation (Ormerod and Galea, 2001). Estrogen plays a decisive role in the formation of dominant follicles. The dominant follicles with high estrogen content and more FSH receptors can further develop into mature follicles, while the follicles with low estrogen synthesis and less FSHR tend to atresia and eventually apoptosis (Onagbesan et al., 2009). In our experiment, we detected the reproductive hormone-associated key genes: *CYP17A1* and *CYP19A1*, and the mRNA expression level of *CYP17A1* in the LWF, SYF and DF was the highest in the 2,700 kcal/kg groups, while *ESR-1* was not changed, suggesting the stimulating effect of dietary energy on *CYP17A1* transcription.

In laying hens, structural bone loss is associated with medullary bone remodeling during Ca mobilization for eggshell formation (Cransberg et al., 2001). Earlier studies have reported that eggshell quality and bone quality are related (Whitehead, 2004; Kim et al., 2012). In the current study, dietary treatments had minimal effects on bone physical characteristics. The finding suggests that the development of femur and tibia is not influenced in the range of tested dietary protein and energy levelsbetween15 wk to 20 wk of age. In previous studies, it is reported that the tibia grows till 25 wk of age (Rath et al., 2000), while different bones responded differently to experimental treatments (Baird et al., 2008; Regmi et al., 2015). The effect of dietary protein and energy level on bone development remains to be elucidated. ALP is an important biomarker for measuring osteoblastic proliferation and bone formation and plays a critical role in bone mineralization (Zhang et al., 2019). In this study, serum ALP activity was similar at the end-of-lay among the different dietary treatment groups indicating a similarity in osteoblastic activity. The results of this study showed that the dietary energy and protein level fed during the prelay period did not affect the BMD and BBS at the end-of-lay, possibly because the prelay diet was fed from 15 to 20 weeks, and during this period, bone formation is targeted towards medullary bone that lacks structural integrity compared to cortical bone (Whitehead, 2004). This is consistent with the observation that dietary protein did not affect bone strength in broiler chickens (Muszyński et al., 2018). Furthermore, the current results corroborate a previous report by Hassan et al. (2013) that BMD and BBS of organic laying hens were not significantly influenced by different dietary energy and protein levels in the diet. Contrary to our result, Jiang et al. (2013) reported that hens fed with high energy diet have lower BMD and BBS. Bone mineralization analysis showed that birds fed with the 15% CP diet had higher tibia ash weight and ash concentration values, likely due to the increase in tibia length in this group. In this study, we observed increased serum P level in 16.5% CP diet. This result was consistent with the observation in broilers by Cowieson et al. (2020), who reported that serum P was decreased by low-protein diet, which is speculated to be a result of reduced dietary P level in low-protein diet. In the present study, the experimental diets had the same amount of P, and hence, the effect of dietary protein level on P metabolism needs to be investigated further.

In conclusion, the results of this study showed that the egg production performance was not changed by the protein and energy levels of prelay diet. Increased dietary CP level in prelay diet increased the egg shape index, eggshell thickness, while the increased energy level increased the yolk pigmentation. The gene expression in the hypothalamus, pituitary, ovary, and follicles of aged hens was differently affected by dietary treatment, whereas the circulating levels of sex hormones were not changed. The long-term effect of prelay diet on the endocrinology of hens during the laying period needs to be investigated further.

## Data Availability

The original contributions presented in the study are included in the article/Supplementary Materials, further inquiries can be directed to the corresponding authors.

## Abbreviations

ADPN, Adiponectin; AgRP, agouti-gelated protein; ALP, alkaline phosphatase; BM, breast muscle; Ca, calcium; CART, cocaine-and amphetamine-regulated transcript; CYP17A1, cytochrome family 17 subfamily A polypeptide 1; CYP19A1, cytochrome P450 family 19 subfamily A polypeptide 1; DF, dominant follicles; DXA, dual-energy x-ray absorptiometry; E_2_, estradiol; FSH, follicle-stimulating hormone; GHRH, growth Hormone Releasing Hormone; GnRH, gonadotropin-releasing hormone and; GnIH, gonadotropin inhibitory hormone; HPG, hypothalamic-pituitary-gonadal; IGF-1, insulin-like growth factor-1; LEPR, leptin receptor; LH, luteinizing hormone; LM, leg muscle; LWF, large white follicles; NPY, neuropeptide Y; OVR, oocyte vitellogenesis receptor; P, phosphorus; P_4_, progesterone; POMC, pro-opiomelanocortin; SIRT1, sirtuin1; SYF, small yellow follicles.
